# Effect of Physical Therapy Modalities on Quality of Life of Head and Neck Cancer Survivors: A Systematic Review with Meta-Analysis

**DOI:** 10.3390/jcm10204696

**Published:** 2021-10-13

**Authors:** Barbara Burgos-Mansilla, Noelia Galiano-Castillo, Mario Lozano-Lozano, Carolina Fernández-Lao, Maria Lopez-Garzon, Manuel Arroyo-Morales

**Affiliations:** 1 Kinesiology Program, Faculty of Health Sciences, Universidad Autónoma de Chile, Avenida Alemania, Temuco 4810101, Chile; barbara.burgos@uautonoma.cl; 2 Department of Physical Therapy, Faculty of Health Sciences, University of Granada, 18016 Granada, Spain; noeliagaliano@ugr.es (N.G.-C.); carolinafl@ugr.es (C.F.-L.); maloga@ugr.es (M.L.-G.); marroyo@ugr.es (M.A.-M.); 3 Sport and Health University Research Institute (iMUDS), 18016 Granada, Spain; 4 Instituto de Investigación Biosanitaria ibs.GRANADA, 18016 Granada, Spain; 5 ‘Cuídate’ Support Unit for Oncology Patients (Bio277 Group), 18016 Granada, Spain

**Keywords:** systematic review, meta-analysis, head and neck neoplasms, cancer survivors, physical therapy modalities, quality of life

## Abstract

The objective was to describe the effectiveness of different physical therapy modalities to improve Quality of Life (QoL) in Head and Neck Cancer (HNC) survivors. PubMed, Scopus, Web of Science, CINAHL and Cochrane Library were searched for randomized clinical controlled trials published until 30 April 2020. Risk of bias assessment and meta-analysis were conducted using the Cochrane tools. A total of 251 records were retrieved, and 10 met the inclusion criteria. Interventions whose parameters focus on a 12-week exercise programs of aerobic activity (walking) or Progressive Resistance Training (PRT) for the whole body are effective and safe modalities improving QoL in HNC survivors. Electrophysical agents did not show significant results between groups. As for the assessment of methodological quality, 4 of the 10 articles included had a high risk of overall bias. Only five articles provided sufficient information to conduct a meta-analysis for exercise program intervention on QoL, showing a tendency in favor of intervention group, even when the global results did not show statistically significant improvements (pooled Cohen’s d 0.11; 95% CI: −0.27 to 0.50; I^2^ 42.68%; *p* heterogeneity = 0.12). The present review and meta-analysis identified meaningful benefits of exercise on QoL of HNC survivors; this has been confirmed in a meta-analysis. This review adds evidence supporting exercise interventions on Head and Neck Cancer population whose opportunities for successful recovery after medical treatment are more limited.

## 1. Introduction

Head and Neck Cancer (HNC) covers sites located on the lips (mucosa surface), oral cavity, pharynx, larynx, cervical esophagus, nose, paranasal sinuses, salivary glands, thyroid gland and parathyroid glands [1]. Both early detection programs and better treatments have been responsible for the steady increase in the survival rate of these patients, in addition to a decrease in smoking habits and better prognosis with human papillomavirus (HPV)-derived cancer [2,3]. For instance, 65.3% of patients diagnosed with HNC in the United States survive 5 years or more [4], and half of people diagnosed with HNC in England survive 10 years or more [5]. HNC mainly affects people of working age, and thus, the economic costs are very high (a 5-year mean cost of USD 79,165 per patient) [6].

A large proportion of these patients receive surgery (32–75%) [5], radiotherapy (RT; 43–85%) [5] and/or chemoradiotherapy (CRT; 8–61%) [5] as part of their primary cancer treatment. Surgery, which is determined by the stage and location of the tumor, presents a diversity of effects according to the timeframe performed, that is, before or after other oncology treatments. Unfortunately, there are adverse effects that remain even 10 years after surgery, such as pain and active trigger points on the head, neck and shoulder muscles, general hypersensitivity and hyperalgesia [7], insomnia and eating problems [8]. In addition, the emotional component of body image is a troublesome factor, considering that the location of these tumors makes them more visible [9]. Conventional 3D RT is related to many delayed impairments, such as trismus, dentition breakdown (radiation caries), loss of salivary gland functions and osteoradionecrosis [10]. Fatigue, emotional distress and low quality of life (QoL) have been described as consequences of intensity-modulated RT [11]. Finally, many HNC patients are treated with CRT (e.g., cetuximab since 2006 in combination with RT). Currently, cisplatin is the most frequently used treatment [12]. CRT implies the greatest disability compared to surgery or RT alone. For example, nutrition impact symptoms such as xerostomia, dysphagia, trismus and oral pain have been described [13,14]. Consequently, all of these biopsychofunctional impairments, due not only to the illness but also to the treatments, have a significant impact on the general QoL of these patients.

“Cancer survivor” has been defined in different ways; one of them is as follows: patients living beyond the end of treatment or 3–5 years from diagnosis in complete remission [15,16].

The survival rate in HNC is one of the most complex owing to the anatomical difficulty of this region [17] and the consequences of medical treatment [18]. Moreover, it is a completely neglected population in terms of rehabilitation strategies [19,20] (if compared to other cancer survivors, such as breast or colorectal cancer). This fact could be due to the short follow-up motivated by nearly 80–90% of all recurrences occurring within the first years. According to Haddad and Limaye [21], there are no data to guide the follow-up of long-term HNC patients, especially head and neck squamous cell carcinoma survivors. The understanding of complications suffered by these patients is based on assessments developed during the first years after treatment completion. That is why there are few and poor studies reporting long-term side effects, which could explain the lack of data on rehabilitation strategies. Specific efforts should be made to design adequate support strategies and rehabilitation programs in this population [22,23].

Physical therapists, who are responsible for examining and managing the side effects derived from cancer and its treatment, have become an indispensable part of the continuum of cancer care [24,25,26]. Different approaches to physical therapy (rehabilitation) might considerably reduce the economic impact of this disease, improving the chances of returning to work [19]. One of the main, widely recognized indicators would be their decrease in terms of QoL perception relating to unemployment or reduced work hours [27]. According to this, QoL could be the outcome that reflects the barriers and interference with daily life experienced by HNC survivors [20].

Between both published reviews and meta-analyses, there is a tendency to evaluate the effects of exercise [28,29,30,31,32,33,34,35,36] in cancer survivors. These interventions have mainly been proven in breast [28,29,30,31] and colorectal cancer [32,33]. With regard to HNC survivors, electrotherapy such as transcutaneous electrical nerve stimulation (TENS) [37,38] and laser therapy [39] have reported promising results on salivary flow rate and QoL. Even acupuncture has shown only a discrete effect, increasing salivary function in patients after RT [40]. Jaw exercises and the use of oral devices have been shown to be useful for mouth interincisal opening in cancer treatment-induced trismus [41]. There are previous systematic reviews that have assessed different interventions in HNC survivors [42,43,44,45,46,47,48,49]; however, some include HNC patients undergoing active treatment as a target population [44,47,48]. None of the mentioned reviews consider QoL as a primary outcome, and most focus on oral and swallowing impairments [43,44,45,46,47,48]. Only the systematic review led by Almeida et al. [42] presents results from studies that measure QoL, but as a secondary outcome. However, [42] uses a valid method and describes that its aim was to assess rehabilitation interventions as a whole and does not compile all potential methods that could be effective in improving QoL. In fact, it was only focused on the assessment of shoulder function. All reports mentioned above, despite the promising findings, present some limitations already described. This highlights the need for an updated review that comprises all these criteria. Therefore, this systematic review aimed to describe the effectiveness of different physical therapy modalities to improve QoL in HNC survivors and to discover which of these modalities would be most effective.

## 2. Materials and Methods

### 2.1. Focused Question

A systematic review protocol that defined inclusion criteria, search strategy and outcomes of interest was developed and registered with PROSPERO (CRD 42020151929, 12 May 2020). Reporting of this systematic review adheres to the Preferred Reporting Items for Systematic Reviews and Meta-Analyses (PRISMA) statement [50]. According to PRISMA guidelines, the specific question posed for the review was, “Are physical therapy modalities effective in improving QoL in HNC survivors? Which physical therapy modalities are most effective in improving the QoL of these patients?”

### 2.2. Search Strategy and Eligibility Criteria

Detailed search strategies were developed for each database used in the review: Medline (via PubMed searcher), Scopus, Web of Science, Cumulative Index for Nursing and Allied Health Literature (CINAHL) and Cochrane Library. The literature search was conducted between 1 March and 30 April 2020. The following keywords were used for the search: “head and neck cancer”, “survivor”, “physical therapy modalities”, “quality of life” (see supplementary material: search strategy). Keywords were combined using the Boolean operators “AND” and “OR”. No restrictions were placed on the year of publication, but only published studies in Spanish and English from inception to 30 April 2020 were considered. Studies were included if they met the following criteria: (1) design: randomized controlled trials (RCTs); (2) population: adults (over 18 years old) considered to be HNC survivors; (3) intervention: physical therapy modalities such as electric stimulation therapy (electroacupuncture, pulsed radiofrequency treatment, transcutaneous electric nerve stimulation), exercise therapy, hydrotherapy, musculoskeletal manipulations (manipulation, motion therapy, massage), myofunctional therapy and laser therapy (low-level light therapy); (4) control group: placebo, usual care or no intervention; and (5) outcome: QoL. Furthermore, an automatic alert notification for new publications relevant to search term combination was created in all databases from the initial search date. Two independent researchers (B.B.M. and M.L.L.) performed the selection of the studies through Covidence systematic review software (Veritas Health Innovation, Melbourne, Australia) [51]; then, the same two reviewers made the final selection of the studies and appraisal of methodological quality. Disagreements were resolved by the judgment of a third author (N.G.C.).

### 2.3. Data Extraction and Quality Assessments

This process was performed independently by two review authors (B.B.M. and M.L.L.) using an Excel spreadsheet applying a predesigned criterion data collection form. The data collected were study characteristics such as authors, country of origin, study design and sample size and participant characteristics such as sex, mean age, stage of cancer (I, II, III or IV), location of HNC (throat, oral, nose, etc.) and type of oncological treatment (radio, chemo, surgery) were included in the data. Additionally, data on the characteristics of the interventions included frequency, duration, comparison, outcome measures, adverse events, measured time points, intervention group (IG), control group (CG), mean change, group differences in mean change and *p*-values. The outcome measures, such as the European Organization for Research and Treatment of Cancer Questionnaire (EORTC QLQ-C30) [52], Functional Assessment of Cancer Therapy General and Head and Neck Module (FACT G and FACT H&N) [53] and Head and Neck Cancer Inventory (HNCI) [54] were recorded. The risk of bias assessment was performed according to the Cochrane Risk of Bias tool: RoB 2 [55].

After the data extraction, the reviewers determined the possibility of performing a meta-analysis by considering if the heterogeneity was moderate or strong as assessed by I^2^ (less than 25%, no heterogeneity; 25–49%, low heterogeneity; 50–74%, moderate heterogeneity; and 75% or greater, high heterogeneity) [56]. For the quantitative combination of the studies, only those that measured QoL by means of a validated instrument presented all of the data necessary to perform it and whose intervention was exercise. With the aim of homogenizing the results, a quantitative combination by subgroups was performed according to the questionnaire used to measure QoL (EORTC QLQ-C30, FACT H&N, FACT G), and forest plots were used to summarize the results. The studies were combined using the random-effects model of the DerSimonian and Laird method, which considers the variations within and between studies, using Cohen’s d effect size as an estimator. The random effects model was used for the analysis. Given the number of included articles (less than 10), it was not possible to perform the publication bias study. For all of the analyses, Stata Statistical Software was used (StataCorp. 2019. Stata Statistical Software: Release 16. StataCorp LLC, College Station, TX, USA).

## 3. Results

**Figure 1:**
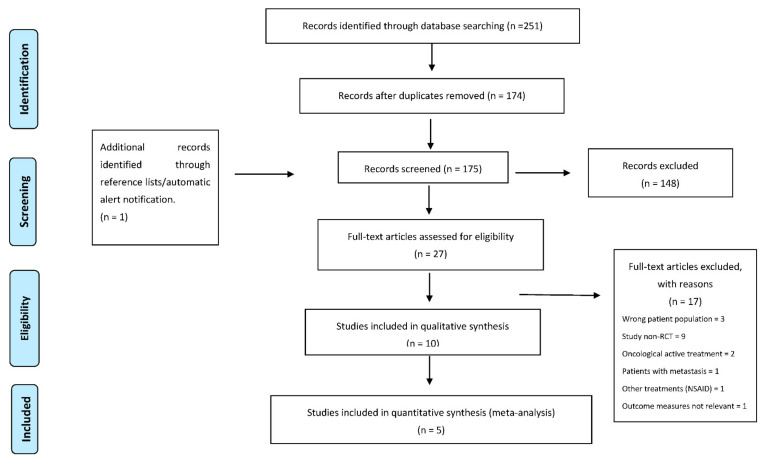
Flowchart of search results and filtering of the documents selected in this study.

The literature search identified 251 articles, with 77 duplicates, and automatic alert notification provided information on approximately 1 new article, which was also included. A total of 148 articles were excluded after screening the titles and abstracts. After that, 27 studies were then retrieved for full-text review, and 17 records were excluded for the following reasons: 3 had incorrect patient populations, 9 were not RCTs, 2 had people undergoing active treatment, 1 included patients with metastasis, 1 included other treatments (nonsteroid anti-inflammatory drugs—NSAIDs) and 1 did not include QoL as a relevant outcome measure. Finally, 10 records were included in this systematic review. Interrater agreement in the selection of studies was 51.4% [57]. After discussion, the reviewers reached consensus (100%). In the PRISMA flowchart, the stages of the review process, including study identification, inclusion and exclusion, are shown (Figure 1).

### 3.1. Descriptive Synthesis

The 10 studies included in this review were conducted across six countries, most commonly in Canada (*n* = 4) [58,59,60,61] and in second place China (*n* = 2) [62,63]. A total of 533 subjects participated in the studies included in this review (292 IG and 241 CG), and most were males (77%). The sample size of the studies ranged between 20 and 170 subjects. The global mean age of all subjects (IG and CG) was 56.4 years, with a range between 48 and 66 years. Analyzing all participants of the included studies, 26.5% were in stage I–II at diagnosis, and 73.5% were in stage III–IV. The most common location of HNC was the pharynx (41%), followed by other sites and the oral cavity. Of the oncological treatments, the most common was surgery and CRT (28%), followed by RT (20%); surgery plus RT was the least common (14%) (Table 1).

Regarding physical therapy modalities, 60% of the studies focused on exercise programs [58,59,62,63,64,65], and 40% of the studies were based on electrophysical agents [60,61,66,67]. Regarding parameters, there was heterogenicity in terms of type of therapy, frequency and global duration (time per session) for both modalities. Considering exercise, three approaches can be distinguished: first, programs based on aerobic exercise [62]; second, programs based on Progressive Resistance Training (PRT) [58,59,64]; and third, a combination of both [63,65]. Regarding parameters for aerobic exercise, one study applied a home-based walking exercise program at a moderate intensity level, 3–5 days per week for 12 weeks for 30 min each session, or a total of 150 min per week [62]. The other two studies [63,65] applied aerobic exercise on a multimodal program through walking with similar parameters. In PRT programs, 2 sets of 8 to 10 repetitions of each exercise [58,64] for 12 weeks were the most commonly used [58,59,63,64,65]. For the muscle groups involved, three studies focused on upper limb and scapular muscle exercises [58,59,63], and the rest focused on the whole body [64,65].

Considering the electrophysical agents, electrostimulation was the main agent performed [60,61,67]. It is important to consider that the aim of electrostimulation differs substantially between studies. One study applied acupuncture-like transcutaneous electrical nerve stimulation (ALTENS) to acupuncture points to improve the salivary flow rate [61]. Two studies used neuromuscular application, one on quadriceps group muscles [60] and the other on suprahyoid muscles [67]. Regarding the parameters of electrostimulation, some similarities were found, with burst modality ranging from 40–70 Hz as the frequency and a width pulse between 80–300 µs. The contraction time varied from 4 to 5 s, a relaxation time of 10–12 s, ramp up 1.5–2 s, ramp down 0–0.75 s and a duration of 20 min. Two out of three studies chose a duration of 12 weeks of intervention [60,67], while the other study used a 6-week intervention time [61]. Only one of four studies applied photobiomodulation therapy (laser therapy) with the following parameters [66]: continuous wave mode, with 830 nm (infrared) wavelength, 100 mW output power, 3.57 W/cm^2^ power density, 71 J/cm^2^ dose per point and application time 20 s on major salivary glands, parotid glands, submandibular glands and sublingual glands (0.028 cm^2^). Patients underwent two weekly sessions for 6 weeks. Of the included studies, only three considered follow-up periods [61,62,65], including 12 weeks to 1 year of follow-up. Regarding CG, one study considered placebo treatment [66], another study used placebo and exercises [67], seven studies applied either usual care or education programs [58,59,60,62,63,64,65], and one study contained three groups of interventions [61]. Finally, QoL was measured with different instruments: FACT G/FACT H&N [58,59,60,63], EORTC QLQ-C30 [62,64,65] and others, such as the Oral Health Impact Profile (OHIP-14) [66], H&N Cancer Inventory [67] and Head and Neck Radiotherapy Questionnaire [61] (Table 2).

### 3.2. Adverse Events

Only one study [58] (10%) reported adverse events in one subject of the IG who experienced pain related to a soft tissue injury on the scapular region. Fifty percent reported no adverse events [59,60,61,64,65], and forty percent [62,63,66,67] did not mention adverse events.

### 3.3. Qualitative Analysis

The analysis was performed by subgroups considering each physical therapy modality. Chang et al. [62] and Lønbro et al. [64] demonstrated an intergroup significant difference in favor of IG on QoL (*p* < 0.05) from baseline to week 12. Both studies used the same instrument (EORTC QLQ-C30). However, O’Neill et al. [65] did not show intergroup significant differences using the same instrument (*p* = 0.433). McNeely et al. [59] and Su et al. [63] measured QoL with FACT H&N, and their results did not show any intergroup significant differences at any time point (*p* > 0.05). Additionally, McNeely et al. [58] did not show intergroup significant differences using FACT G. Concerning intragroup results, only Chang et al. [62] and Lønbro et al. [64] had favorable results for both the IG (*p* < 0.001) and CG (*p* < 0.05) postintervention. Considering studies that tested electrophysical agents, no intergroup differences were found (*p* > 0.05) [60,61,66,67]; however, the majority of the studies [60,66,67] showed favorable intragroup results for both IG and CG at different time points (*p* < 0.05), although different instruments were used as outcomes (H&N Cancer Inventory, OHIP-14, FACT H&N). Regarding follow up, only three studies assessed cumulative effects [61,62,65] as it was described previously, but none of the studies found effects maintained over time (Table 2).

### 3.4. Risk of Bias in the Included RCTs

**Figure 2:**
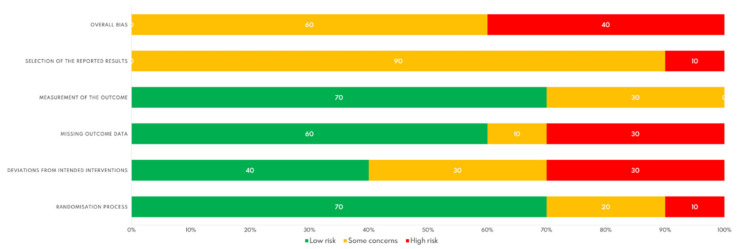
Risk of bias graph.

The results of the methodological quality assessment of the 10 included RCTs are shown in Figure 2.

**Figure 3:**
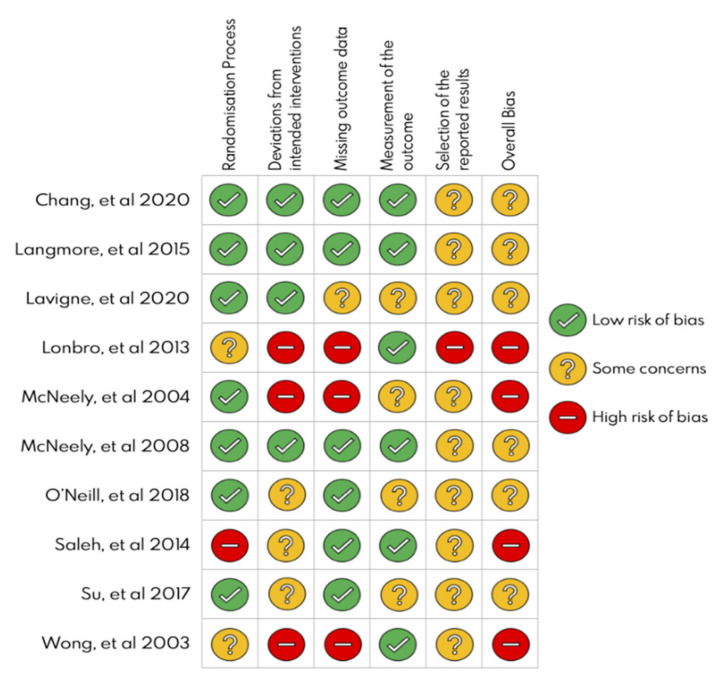
Risk of bias summary of the included studies.

The major methodological quality issues were deviations from intended interventions (30%) and missing outcome data (30%) with “high risk”. In contrast, “low risk” percentages were reported for the randomization process (70%) and measurement of the outcome (70%). Figure 3 shows an assessment summary for each study. All the studies included in this systematic review failed (partial or totally) in the selection of the reported result. Therefore, none of the studies achieved a “low” overall risk of bias.

### 3.5. Meta-Analysis

Of the ten studies included in the systematic review that measured QoL, it was only possible to include five in the meta-analysis [58,59,63,64,65]; the study by Chang et al. [62] was excluded, as it did not present the necessary data for extraction in the meta-analysis.

The meta-analysis included a total of 182 participants, 93 (IG) and 89 (CG). Regarding the assessment instruments used to measure QoL, two studies used the EORTC QLQ-C30 [64,65], two others used the FACT H&N [59,63] and McNeely et al. [58] used the FACT G. McNeely et al.’s [59] study also measured QoL with the latter questionnaire, so its data were used for pooling. In the case of the EORTC QLQ-C30, the items are scored on a Likert scale of four points and have subscales (functioning, symptoms and global health). Higher scores on the functioning subscales and global health status reflect better health conditions; in contrast, higher scores on symptom subscales show critical symptoms, and finally, the subscale scores are transformed to a scale from 0 to 100 [52]. The FACT G has 27 items and also uses a Likert scale, but of five points; the subitems of this questionnaire are physical well-being, social/family well-being, emotional well-being and functional well-being. Higher scores mean better conditions, and by adding the subitems, the total score of the FACT G ranges from 0 to 108 points. The FACT H&N has 39 items (27 of FACT G and 12 more of specific symptoms); on all of the instruments, higher scores are related to better QoL [53].

**Figure 4:**
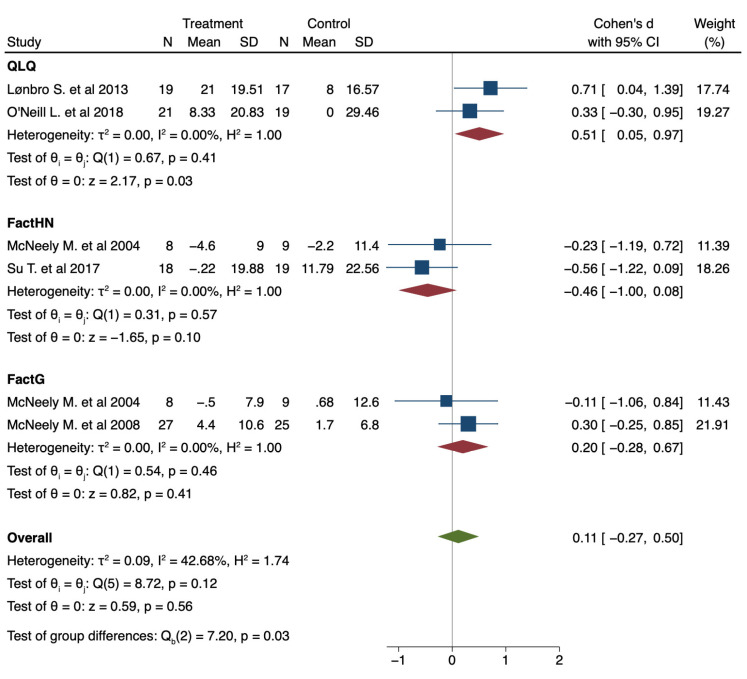
Forest plot presenting the effect of exercise on the improvement of Quality of Life (QoL) measured with different instruments in patients with Head and Neck cancer (HNC) compared with control; pre–post intervention data. Values on x-axis denote Cohen’s d. The diamond illustrates the 95% confidence interval of the pooled effects.

Regarding the data presented, there seems to be a tendency in favor of IG in terms of improvement in QoL after exercise program intervention (pooled Cohen’s d 0.11; 95% CI: −0.27 to 0.50; I^2^ 42.68%; *p* heterogeneity = 0.12). Pooled results are presented in Figure 4.

## 4. Discussion

The main findings of this systematic review were that 12-week exercise programs focusing on aerobic activity (walking) or PRT for the whole body seem to be the modalities with more benefits to ameliorate QoL perception in HNC survivors. Neither electrophysical agent (electrical stimulation or laser therapy) showed significant results between groups, although almost every group improved their results. The meta-analysis supports the results in favor of exercise programs.

HNC survivors are recognized as a heterogeneous population in regard to the location of the tumor [9], and as a troublesome area of the body, it would be interesting to describe which programs involving aerobic and/or resistance exercises might be recommended [62,64]. The rest of the approaches did not detect differences in QoL, which could be related to the instrument used to measure QoL [58,59,63,65]; it is known that the EORTC QLQ-C30 is the most widely used multidimensional assessment of health-related quality of life [52]. Oneill et al. [65] stated that, although EORTC QLQ-C30 was used in their assessment, surprisingly, improvements were not found following their intervention. This instrument may not be the most suitable to detect subjective benefits described by their participants (e.g., confidence, social functioning). The findings defend the EORTC QLQ-C30 as an overall instrument able to demonstrate the effect of different modalities of exercise, particularly in a population dealing with numerous treatment-related morbidities [7,8,9,10,11,12,13]. The rest of the instruments, which are presumably more specific (e.g., FACT H&N) [53], may have been less sensitive to the effect of the mentioned approaches [58,59,63]. Exercises that involved the whole body have been used, and it seems logical to believe that changes in overall QoL would be desirable; hence, a global instrument such as the EORTC QLQ-C30 should be used in future studies. Additionally, there were differences between the studies of this review on frequency and intensity of the PRT [58,59,63,64,65]. Although guidelines on this matter are general for survivors of cancer [68,69], parameters of doses/response could be a future line of research.

On the one hand, there are reports that involved only upper limb and scapular muscles [58,59,63], and the fact that Lønbro et al. [64] also involved spine muscles could have been decisive. All exercise programs described in this systematic review focused on strengthening the whole body (upper and lower limbs and even spine muscles), although the most popular locations in this review were the pharynx and oral cavity [58,59,63,64]. A possible explication could be that sensitive disorders caused by both cancer and treatment can determine this selection of global intervention instead of others more specifically [7,70,71]. Other low-intensity exercises whose target is the oral and cervical regions may have reported better results in terms of pain, which could translate to better QoL [7]. The potential mechanism responsible for these changes could be the increases of muscle mass, muscle strength and functional performance [68]. Although the search strategy was complex, other oral–cervical-based modalities, such as massage, were missed in this review if the majority of patients retrieved underwent a process of surgery that supposes an important physical cost, themselves [72,73,74].

Another detail that could have been crucial would be the use of an informatics component [62]. Monitoring compliance plus guidelines for diet and lifestyle changes are key challenges, particularly in survivors of cancer where habitual exercise participation is limited [75,76]. In contrast and, according to this review, an excellent adherence rate (approximately 93%) was registered in all exercise programs [58,59,62,63,64,65]. A proposal for active telerehabilitation, based on feedback technology and face-to-face contact and designed with patients’ perspectives in mind, would be more appropriate to involve patients in treatment [77,78] and possibly reach a greater level of significance.

Finally, all reports were performed over 12 weeks (with or without significance), so it seems to be an adequate point to observe benefits in terms of QoL. Both modalities (aerobic and/or resistance exercises) should be considered in the rehabilitation of HNC survivors. Research within breast and colon cancer populations has already demonstrated relevant effects of different exercise interventions on global health status, pain, QoL and fatigue through clinical trials [79,80,81,82,83,84]. Additionally, the results are coincident with other reviews on breast and colon cancer patients [28,29,30,31,32,33], considering that this intervention should be initiated as soon as possible in the early posttreatment period, even from diagnosis, as the literature increasingly supports it [25,85].

Regarding electrophysical agent studies, there was no benefit between groups, even when the intervention and comparison groups were successfully separated [60,66,67]. Several explanations could be suitable: basic oral hygiene given all patients [66] could be responsible for improvements due to the fact that, although its evidence is limited, it is known that a basic self-care protocol might achieve certain symptomatic relief [86]. Similar justification could be behind the improvement described by Lavigne et al. [60], as the use of an exercise CG within trial would justify benefits in both groups. Other work led by Langmore et al. [67] showed disappointing results on their main outcome (swallowing function); however, all their patients reported better QoL, which could be due to a simple placebo effect of participation in clinical trials [87]. Wong et al. [61] reported benefits in xerostomia symptoms throughout different protocols of ALTENS, but this did not result in better QoL. The authors suggest that a well-designed, placebo-controlled trial should be undertaken to further evaluate their hypothesis; however, some of these authors explained years later that appropriate sham control remains methodologically challenging for this modality [88]. Although three out of four studies reported the usage of electrical stimulation (neuromuscular and meridian-based points), the heterogeneity of parameters was decisive to complicate a consensus. A recent review with meta-analysis suggests that laser therapy is an effective, noninvasive and safe approach in patients with xerostomia (cancer and no cancer) [89]. However, the lack of significant results described by Saleh et al. [66] could have been related to the late effects of RT on glandular structure and its permanent damage over time [90] due to patients were treated at least 6 months after RT, where the potential benefits of laser could be lower.

Meta-analysis suggests that patients in the exercise group improved their QoL compared to the CG. Regarding the results and the aim of systematic review, the possibility of carrying out a network meta-analysis was raised to determine the mediation effect between the different physical therapy interventions proposed in the global effect on QoL and to establish the best possible treatment. However, although it would have been interesting to know a global estimator of the efficacy of these interventions in improving the QoL of HNC, the heterogeneity of these interventions, added to the small number of studies collected, made it impossible to combine them quantitatively. However, this same reasoning supports the results of the meta-analysis in favor of exercise: the inconclusive results of the other techniques and the low number of studies, together with the significant tendency shown in the meta-analysis, seem to postulate exercise as the gold standard in improving the QoL of these patients. 

The results are in line with several meta-analyses [77,91,92,93] studying the effect of exercise in different cancer populations, mainly breast cancer, all of which showed results in favor of the intervention group. Finally, the low or null statistical heterogeneity observed in the subgroup analysis carried out is noteworthy, which reinforces the results of the meta-analysis, despite the small number of studies included. Although it is true that this heterogeneity is moderate in the overall analysis, this is explained by the variability of the assessment instrument used to measure QoL, which the choice of the statistical method used is intended to counteract.

To our knowledge, this is the first review evaluating the effect of different physical therapy modalities on improving QoL in HNC survivors. The strengths of this review are reporting according to the PRISMA guidelines, inclusion of risk of bias assessment and meta-analysis with the low statistical heterogeneity obtained.

### Study Limitations

Limitations in published reports restricted the ability to determine those parameters of the interventions that were effective; further limitations include that the meta-analysis comprises only five studies and none of studies achieved a low overall risk of bias assessment, and it was not possible to combine the data from the studies that used electrophysical agents due to lack of methodological similarity between them.

## 5. Conclusions

In summary, this systematic review identified meaningful benefits of exercise with regard to QoL. This has been confirmed in a meta-analysis that comprises five out of ten studies involved. However, reports based on electrophysical agents such as electrostimulation and laser therapy were not able to find results between groups despite groups improving separately. The maintenance of effects in both modalities was also inconclusive. The potential of exercise and partly electrophysical agents to enhance QoL in HNC survivors seems to be clear, although it has not been possible to reach a reliable consensus in terms of the parameters analyzed due to the disparity in the data retrieved. This systematic review has brought to light the gaps in physical therapy strategies that affect this oncology population. This issue can be a starting point for future lines of research. To reinforce the emerging findings, it has found that all methods evaluated seem to be safe. This review adds to the growing evidence supporting exercise interventions to improve QoL in a cancer population such as HNC survivors, whose opportunities for successful recovery after medical treatment are more limited.

## Figures and Tables

**Table 1:** Descriptive Synthesis of the included studies.

Authors	Country	Study Design	Sample Size	Gender (% Male)	Mean Age (Years)	Stage of Cancer at Diagnosis (%)		Location of HNC (%)					Oncological Treatment (%)						
						I–II	III–IV	Pharynx	Larynx	Oral Cavity	Lip	Others	RT	CT	S	CRT	S + RT	S + CT	RT + S+CT
Chang et al., 2020	China	RCT	88	91%	56	*	*	20	0	0	0	80	0	0	100	0	0	0	0
Langmore et al., 2016	United States	RCT	170	86%	61.9	15	85	70	12	8	0	10	29	0	0	71	0	0	0
Lavigne et al., 2020	Canada	RCT	22	64%	52	45	55	59	5	18	0	18	0	82	18	0	0	0	0
Lønbro et al., 2013	Denmark	RCT	41	66%	57	20	80	73	2	7	0	17	49	51	0	0	0	0	0
McNeely et al., 2004	Canada	RCT	20	82%	61	18	82	47	29	12	0	12	0	0	6	0	94	0	0
McNeely et al., 2008	Canada	RCT	52	71%	52	18	82	62	23	0	0	15	0	0	0	0	75	25	0
O’Neill et al., 2018	Ireland	RCT	43	81%	66	49	51	0	0	0	0	100	0	0	100	0	0	0	0
Saleh et al., 2014	Brazil	RCT	23	65%	57	17	83	*	*	*	*	*	0	0	0	26	43	0	30
Su et al., 2017	China	RCT	37	92%	48	30	70	0	0	81	0	19	30	43	27	0	30	43	0
Wong et al., 2003	Canada	RCT	37	75%	59	*	*	*	*	*	*	*	100	0	0	0	0	0	0

HNC: Head and Neck Cancer, RT: radiotherapy, CT: chemotherapy, S: surgery, CRT: chemoradiotherapy, RCT: randomized controlled trial, *: not reported.

**Table 2:** Summary of the interventions on the included studies.

Exercise														
Author		Sample Size	Intervention	Frequency	Duration	Comparison	Outcome Measures	Adverse Events	Measured Time Points	IG (Mean, SD)	CG (Mean, SD)	Mean Change (Mean, SD)	Group Differences in Mean Change: Mean (95% CI)	*p*-Values
Chang et al., 2020		44 IG 44 CG	Walking exercise and nursing education health informatics program	3–5 days per week for 30 min each time, or a total of 150 min per week (from 55% to 65% of HRR)	12 weeks	Usual Care	EORTC QLQ-C30	Not reported	Baseline, week 4, week 12, 3-month follow-up	Baseline: 47.5 (5.64)	Baseline: 44.5 (8.50)	Not reported	Not reported	**Within groups** Baseline–week 4: IG and CG *p* < 0.001 Baseline–week 12: IG and CG *p* < 0.001 Baseline–3-month follow-up: IG and CG *p* < 0.001 **Between groups (IG vs. CG)** Week 4: IG > CG ***p* < 0.05**
Lønbro et al., 2013		20 IG 21 CG	PRT: leg press, knee extension, hamstring curls, chest press, sit ups, back extensions and lateral pull down.	2–3 sets of 8–15 RM of 7 exercises. 2 to 3 sessions per week. 30 sessions total	12 weeks	Usual Care	EORTC QLQ-C30	No	Baseline and week 12	Baseline: 53 ± 19 Week 12: 74 ± 20	Baseline: 70 ± 15 Week 12: 78 ± 18	Baseline–week 12: IG: 19 ± 14//CG: 6 ± 12	Not reported	**Within groups** Baseline–week 12: IG *p* < 0.001/CG *p* < 0.05 **Between groups (IG vs. CG)** Baseline–week 12: IG >CG ***p* < 0.05**
McNeely et al., 2004		10 IG 10 CG	PRT on upper limbs and scapular muscles	1-2 sets of 15 to 25 RM of 6 exercises. 3 times per week	12 weeks	Usual Care	FACT H&N	No	Baseline and week 12	Baseline: 109.5 (12.2) Week 12: 104.8 (18.5)	Baseline: 103.1 (22.4) Week 12: 100.9 (23.9)	CG: −2.2 (11.4) IG: −4.6 (9.0)	−2.4 (−13.2 to 8.3)	**Within groups** Baseline–week 12: IG and CG *p* > 0.05 **Between groups (IG vs. CG)** Baseline–week 12: *p* = 0.639
McNeely et al., 2008		27 IG 25 CG	PRT on upper limbs and scapular muscles	2 sets of 10 to 15 repetitions of 5 to 8 exercises. Between 25% at initial and 70% at the end of the program of the 1-RM, 3 times per week	12 weeks	Usual Care	FACT G	Pain	Baseline and week 12	Baseline: 79.4 (13.7) Week 12: 83.9 (15.6)	Baseline: 76.4 (18.4) Week 12: 78.1 (19.3)	CG: +1.7 (6.9) IG: +4.4 (10.6)	+4.5 (−0.7 to 9.7)	**Within groups** Not reported **Between groups (IG vs. CG)** Baseline–week 12: *p* = 0.091
O’Neill et al., 2018		21 IG 22 CG	Aerobic: walking, stationary cycling and cross training PRT: upper and lower limb muscles	Aerobic: 3 to 5 days per week (from 30% of HRR at initial weeks to 60% of HRR at the end of the program)PRT: twice a week (from 2 sets/muscle groups at initial weeks to 6 sets/muscle groups at the end of the program)	12 weeks	Usual Care	EORTC QLQ-C30	No	Baseline, immediately postintervention and at 3-month follow-up	Baseline: 75.00 (20.83) Week 12: 83.33 (20.83) 3-month follow up: 79.17 (29.16)	Baseline: 66.67 (33.33) Week 12: 66.67 (25.00) 3-month follow up: 75.00 (16.6)	Not reported	Not reported	**Within groups** Not reported **Between groups (IG vs. CG)** Baseline–week 12: *p* = 0.433 Baseline–3-month follow-up: *p* = 0.887
Su et al., 2017		18 IG/19 CG	HBP Aerobic: walking PRT: upper limb muscles	Once a day/5 days per week Aerobic: 50 min PRT: 2 sets/10 repetitions/muscle group	12 consecutive weeks	OPT: Aerobic: Walking was performed on treadmill. No PRT	FACT H&N	Not reported	Baseline, week 6, week 12	Baseline: 93.83 (19.73) Week 6: 94.89 (22.44) Week 12: 93.61 (21.487)	Baseline: 91.63 (23.59) Week 6: 95.21 (22.27) Week 12: 103.42 (20.02)	Not reported	Not reported	**Within groups** Baseline–week 12: IG and CG *p* > 0.05 **Between groups (IG vs. CG)** Baseline–week 12: *p* = 0.074
**Electrophysical Agent**														
**Author**	**Sample Size**	**Intervention**	**Parameters**	**Frequency**	**Time**	**Comparison**	**Outcome Measures**	**Adverse Events**	**Measured Time Points**	**QoL IG (Mean, SD)**	**QoL CG (Mean, SD)**	**Mean Change (Mean, SD)**	**Group Differences in Mean Change: Mean (95% CI)**	***p*-Values**
Wong et al., 2003	Group A: 13 Group B: 10 Group C: 14	ALTENS Group A: Sp6, St36, LI4 (active electrodes) and CV24 (indifferent electrode)	Nonpolarizing, balanced, biphasic, square electrical pulses of 250-ms. Trains with a repetition rate of 4 Hz. Each acupuncture point was randomly stimulated for 10 s each time	Twice weekly	6 weeks	ALTENS Group B: Sp6, St36, P6 (active electrodes) and CV24 (indifferent electrode) Group C: Sp6, St5 and 6, P6 (active electrodes) and CV24 (indifferent electrode)	Head and Neck Radiotherapy Questionnaire	No	Baseline and 6, 8 and 12 weeks after treatment began and at 3, 6 and 12 months after treatment completion.	Not reported	Not reported	Not reported	Not reported	**Within groups** Not reported **Between groups (IG vs. CG)** Baseline–6 month follow-up: *p* > 0.05
Saleh et al., 2014	12 IG/11 CG	Laser therapy Application on major salivary glands, parotid, submandibular and sublingual glands	Continuous wave mode. 830nm (infrared) wavelength, 100 mW output power, 3.57 W/cm^2^ power density, 71 J/cm^2^ dose per point, 2 J energy per point, application time 20 sec per point and 28 J dose per session. The area of the spot was 0.028 cm^2^	Twice a week	6 weeks	Sham laser therapy	OHIP-14	Not reported	Baseline, 6th session, 12th session	Baseline: 10.48 (6.82–14.00) 6th session: 7.55 (5.65–11.19) 12th session: 2.5(1.69–9.84)	Baseline:10.23 (6.39–12.82) 6th session: 5.17 (2.28–10.69) 12th session: 3.53 (0.66–10.44)	Not reported	Not reported	**Within groups** Baseline–12th session IG and CG *p* < 0.05 **Between groups (IG vs. CG)** Baseline, *p* = 0.786 6th session, *p* = 0.413 12th session, *p* = 0.976
Langmore et al., 2016	116 IG/54 CG	E-stim device: Electrical Stimulation to stimulate the suprahyoid muscles + swallow exercises. 5-minute warmup stretching protocol followed by swallowing 60 times in synchrony with the stimulation	Frequency 70 Hz Pulse width 300 microseconds (range, 130–300) Contraction 4 s (range, 4–8) Relaxation 12 s (range, 12–16) Ramp up 2 s (range, 2–4) Ramp down 0 s Amplitude limit 0–99 Treatment time 20 min or longer if needed	Twice per day, 6 days per week	12 weeks	Sham device+ swallow exercises. 5-minute warmup stretching protocol followed by swallowing 60 times in synchrony with the stimulation	HNCI	Not reported	Week 7 and week 12	Baseline: 32.54 (21.04) Week 12: 38.85 (23.97)	Baseline: 24.18 (18.58) Week 12: 30.93 (20.46)	IG: 6.31 (17.92) CG: 6.74 (15.59)	HNCI Speech:−3.37 (29.81 to 3.06) HNCI eating 1.41 (25.28 to 8.10) HNCI aesthetics 0.49 (27.98 to 8.95) HNCI social disruption −3.11 (210.28 to 4.05)	**Within groups** Baseline–week 12 IG: HNCI speech: *p* = 0.016 HNCI eating: *p* < 0.001 CG: HNCI speech: *p* = 0.001 HNCI eating: *p* = 0.003 **Between groups (IG vs. CG)** Baseline–week 12 HNCI Speech: *p* = 0.304 HNCI Eating: *p* = 0.679 HNCI aesthetics: *p* = 0.910 HNCI social disruption: *p* = 0.395
Lavigne et al., 2020	11 IG/11 CG	NMES and eccentrically overloaded unilateral squats	2 sets × 8 repetitions of unilateral squats. Negative electrode over the femoral triangle of each leg, 1–3 cm below the inguinal ligament. The positive electrodes over the vastus lateralis and distally over the vastus medialis of each leg Frequency: 40 Hz Pulse duration: 180 μs. Contraction–relaxation period: 5 s/10 sRamp-up time: 1.5 s Ramp-down time: 0–75 s	Three times per week	12 weeks	Conventional strength training	FACT H&N	No	Baseline and week 12	Baseline: 116 (18) Week 12: 126 (14)	Baseline: 103 (17) Week 12: 122 (13)	IG: 10 (9) CG: 18 (9)	Not reported	**Within groups** Baseline–week 12 IG and CG *p* = 0.001 **Between groups (IG vs. CG)** Baseline–week 12 *p* > 0.05

QoL: Quality of Life, IG: Intervention Group, SD: standard deviation, CG: control group, CI: confidence interval, HRR: Heart Rate Reserve, EORTC QLQ-C30: European Organization for Research and Treatment of Cancer Questionnaire, PRT: Progressive Resistance Training, RM: Repetition Maximum, FACT G: Functional assessment of cancer therapy—general, HBP: Home Based Program, OPT: Outpatient Physical Therapy, FACT H&N: Functional assessment of cancer therapy—head and neck module. ALTENS: Acupuncture-Like Transcutaneous Electrical Nerve Stimulation, OHIP-14: Oral Health Impact Profile, HNCI: Head and Neck Cancer Inventory, NMES: neuromuscular electrical stimulation.

## Data Availability

Not applicable.
