# Correction: Burgos-Mansilla et al. Effect of Physical Therapy Modalities on Quality of Life of Head and Neck Cancer Survivors: A Systematic Review with Meta-Analysis. *J. Clin. Med.* 2021, *10*, 4696

**DOI:** 10.3390/jcm12030827

**Published:** 2023-01-20

**Authors:** Barbara Burgos-Mansilla, Noelia Galiano-Castillo, Mario Lozano-Lozano, Carolina Fernández-Lao, Maria Lopez-Garzon, Manuel Arroyo-Morales

**Affiliations:** 1 Kinesiology Program, Faculty of Health Sciences, Universidad Autónoma de Chile, Avenida Alemania, Temuco 4810101, Chile; 2 Department of Physical Therapy, Faculty of Health Sciences, University of Granada, 18016 Granada, Spain; 3 Sport and Health University Research Institute (iMUDS), 18016 Granada, Spain; 4 Instituto de Investigación Biosanitaria ibs.GRANADA, 18016 Granada, Spain; 5 ‘Cuídate’ Support Unit for Oncology Patients (Bio277 Group), 18016 Granada, Spain

## Error in Figure

In the original publication [1], there was a mistake in Figure 4 as published. Some standard deviations were wrong. The corrected Figure 4 appears below.

## Text Correction

There was an error in the original publication [1]. Two sentences, one in the summary and one in the results, have an error in the hundredths of some data.

A correction has been made to Abstract section, the seventh sentence and Section 3.5, third paragraph.

The correct text of Abstract section, the seventh sentence is as follows: …showing a tendency in favor of intervention group, even when the global results did not show statistically significant improvements (pooled Cohen’s d 0.11; 95% CI: −0.27 to 0.50; I^2^ 42.68%; *p* heterogeneity = 0.12).

The correct text of the Section 3.5, third paragraph is as follows: Regarding the data presented, there seems to be a tendency in favor of IG in terms of improvement in QoL after exercise program intervention (pooled Cohen’s d 0.11; 95% CI: −0.27 to 0.50; I^2^ 42.68%; *p* heterogeneity = 0.12).

The authors state that the scientific conclusions are unaffected. This correction was approved by the Academic Editor. The original publication has also been updated.

## Figures and Tables

**Figure 4:**
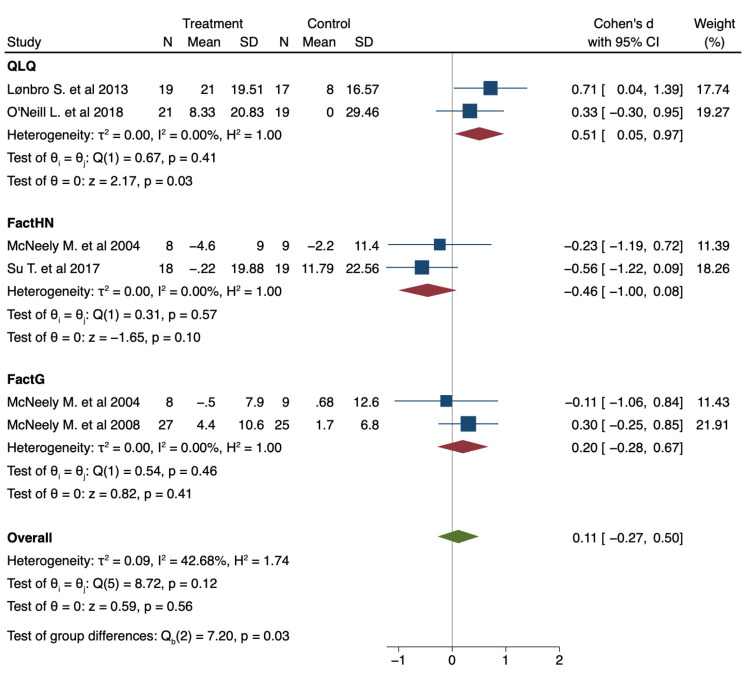
Forest plot presenting the effect of exercise on the improvement of Quality of Life (QoL) measured with different instruments in patients with Head and Neck cancer (HNC) compared with control; pre–post intervention data. Values on x-axis denote Cohen’s d. The diamond illustrates the 95% confidence interval of the pooled effects.
